# Low back pain beliefs are associated to age, location of work, education and pain-related disability in Chinese healthcare professionals working in China: a cross sectional survey

**DOI:** 10.1186/1471-2474-15-255

**Published:** 2014-07-28

**Authors:** B-K Tan, Anne J Smith, Peter B O’Sullivan, Gang Chen, Angus F Burnett, Andrew M Briggs

**Affiliations:** 1 International Health, School of Nursing and Midwifery, Curtin University, GPO Box U 1987, Perth, Western Australia 6845, Australia; 2 School of Physiotherapy & Exercise Science, Curtin University, GPO Box U 1987, Perth, Western Australia 6845, Australia; 3 School of Public Health, Fudan University, 138 Yi Xue Yuan Road, Shanghai 200032, PR China; 4 School of Exercise and Health Sciences, Edith Cowan University, Joondalup, Western Australia, Australia; 5 ASPETAR, Qatar Orthopaedic and Sports Medicine Hospital, Doha, Qatar; 6 Arthritis and Osteoporosis Victoria, PO Box 130, Caulfield South 3162 Victoria, Australia

**Keywords:** Low back pain, Back pain beliefs, China, Healthcare professionals, Fear avoidance

## Abstract

**Background:**

Low back pain (LBP) is the leading cause of disability worldwide. Evidence pointing towards a more efficacious model of care using a biopsychosocial approach for LBP management highlights the need to understand the pain-related beliefs of patients and those who treat them. The beliefs held by healthcare professionals (HCPs) are known to influence the treatment advice given to patients and consequently management outcomes. Back pain beliefs are known to be influenced by factors such as culture, education, health literacy, place of work, personal experience of LBP and the sequelae of LBP such as disability. There is currently a knowledge gap among these relationships in non-western countries. The aim of this study was to examine the associations between LBP-related beliefs among Chinese HCPs and characteristics of these HCPs.

**Methods:**

A convenience sample of 432 HCPs working in various health settings in Shanghai, China, completed a series of questionnaires assessing their demographic characteristics, LBP status, pain-related disability and their beliefs about their own LBP experience, using the Back beliefs Questionnaire (BBQ) and the Fear Avoidance Beliefs Questionnaire (FABQ).

**Results:**

Younger Chinese HCPs (20–29 years) held more negative beliefs and attitudes related to LBP compared to older HCPs (>40years; BBQ mean difference [95% CI]: 2.4 [0.9 - 3.9], p = 0.001). HCPs working outside tertiary hospitals had poorer beliefs concerning the inevitable consequences of LBP (BBQ mean difference [95% CI]: −2.4 [−3.8 - −1.0], p = 0.001). HCPs who experienced LBP had higher level of fear avoidance beliefs when experiencing high LBP-related disability (FABQ-physical mean difference [95% CI]: 2.8 [1.5 - 4.1], p < 0.001; FABQ-work mean difference [95% CI]: 6.2 [4.0 - 8.4], p < 0.001)) and had lower level of fear avoidance beliefs if they had completed postgraduate study (FABQ-physical mean difference [95% CI]: 2.9 [−5.8 - 0.0], p = 0.049).

**Conclusion:**

This study suggests that LBP-related beliefs and attitudes among Chinese HCPs are influenced by age, location of work, level of LBP-related disability and education level. Understanding back pain beliefs of Chinese HCPs forms an important foundation for future studies into the condition and its management in China.

## Background

Low back pain (LBP) is recognized as a leading cause of disability in working populations [1-4]. The Global Burden of Disease Study 2010 identified LBP as one of the leading causes of disability worldwide [4,5]. In 2010, based on disability-adjusted life years as a measure of disease burden, LBP was ranked 6^th^, a concerning ascend, compared to its 11^th^ place in 1990 [5]. Some occupational groups are at an increased risk of developing LBP due to the nature of their duties. For example, healthcare professionals (HCPs) are known to have a high incidence of LBP; the lifetime LBP prevalence amongst rheumatologists and physiotherapists is known to be 46% [6] and 74% [7], respectively. The LBP prevalence rates among nurses, from varying ethnic backgrounds, experiencing LBP during a 12-month period have been reported to range from 56% to 90% [8-13]. Similar occupation-specific trends have also been identified for thoracic spine pain [14]. The significant personal and community economic consequence of LBP such as the loss of productivity, absenteeism and early attrition from the workforce [15-17] highlight the need to identify solutions to resolve this health problem in working populations [18].

The biopsychosocial model of care for management of chronic LBP focuses on effective self-management and capacity of the consumer to actively engage in co-management of the experience [19,20]. Self-management behaviours may be influenced by beliefs and attitudes associated with the experience of LBP and its consequences. Indeed, it has been shown that unhelpful beliefs are associated with higher levels of pain and disability [21-24]. These beliefs can be influenced either positively or negatively by interactions with HCPs [25-27], which are in turn influenced by health professionals’ training, their clinical interests in the management of LBP and the beliefs held by the HCPs themselves [27-29].

The investigation of LBP beliefs held by HCPs has been the focus of recent research as they are known to affect treatment advice given [6,27,30-32] and can influence the beliefs of their patients [25,26,33]. Importantly, these beliefs may contribute to the development of chronic LBP disorders, related disability, care seeking and self-management behaviours [24,30,34]. Understanding the LBP-related beliefs of HCPs and their drivers is, therefore, important for the development of professional education and supporting consumers in effective co-management.

Back pain beliefs are known to be affected by factors such as culture, education, health literacy, place of work, personal experience of LBP and the sequelae of LBP such as disability [24,35-38]. For HCPs, the organizational and professional settings, experience in clinical practice and educational history are important correlates, some of which are potentially modifiable [39,40]. Most research on pain beliefs have, however, been almost exclusively investigated in developed western countries, creating a knowledge gap in this area in other ethnic groups. Chinese culture has been considered to influence health beliefs related to chronic diseases [41-43]. A recent study showed that Taiwanese and Singaporean-Chinese undergraduate physiotherapy and nursing students displayed more negative beliefs about the inevitable consequences of LBP and were more fear avoidant compared to their Caucasian Australians counterparts [35]. Understanding back pain beliefs in Chinese HCPs is, therefore, important due to the increase in migration trends of Chinese HCPs to western countries [44] and the potential influence they bring to their clinical practice in new host country; the rapid modernization of China with the adoption of western culture and; beliefs linked to changes in health policy and practice in that nation.

To date, LBP research in China has focused on prevalence data and on physical and environmental risk factors [45-48] and there has been no research examining back pain beliefs in the Chinese population. This is despite there being a growing interest in the related literature in back pain beliefs in patients, general population and HCPs. Therefore, the aim of this study was to examine the LBP-related beliefs, including beliefs surrounding the inevitable consequences of back pain and fear-avoidance attitudes related to their personal experience of LBP, among Chinese HCPs and to explore the association between these beliefs and characteristics of the HCPs including, location of work, professional discipline, history of LBP and LBP-related disability.

## Methods

### Study Design

A cross-sectional survey, using convenience samples.

### Research settings

Chinese HCPs working in Shanghai, China, were recruited for this study. In 2012, Shanghai was recorded to have a population of 23 million residents, representing China’s largest city [49]. To investigate whether location of work/professional setting influences back pain beliefs, data were collected sequentially in two locations; HCPs working in tertiary teaching hospitals in metropolitan Shanghai (Xuhui, Jing’An and Yangpu districts) and HCPs working in community health centres in Jin Shan district. The larger community health centres in Jin Shan district have both inpatient and outpatient departments. The Jin Shan district is 69 km southwest from Shanghai city and relative to the metropolitan city of Shanghai represents a mixed urban and rural area. It is the second furthest district from metropolitan Shanghai, not serviced by Shanghai’s extensive metro sub-way system. In 2011, Jin Shan had an urban population of 337 340 and a rural population of 179 466 [50]. The per capita annual disposable income of the urban and rural residents of Jin Shan was 28 640 yuan (≈US$ 4 700) and 14 199 yuan (≈US$2 300), respectively [50]. In metropolitan Shanghai, urban residents have an annual per capita disposable income of 40 188 yuan (≈US$6 600) [49]. The key differentiators for the two areas are urban versus urban–rural mix and the care settings in which the professionals worked (tertiary hospitals in Shanghai metropolitan districts versus community health centres in Jin Shan district).

### Participants and recruitment

In this study, the term HCPs refers to any professional working in a government funded hospitals or health settings who are involved in providing patient care. HCPs working in these settings would be professionally qualified to practice in their respective fields.

A convenience sample of registered medical practitioners, traditional Chinese medicine practitioners, therapists (general rehabilitation therapists or therapists in specialised fields such as physiotherapists or occupational therapists) and nurses working in three main tertiary hospitals in metropolitan Shanghai, and 11 community health centres in Jin Shan district, were recruited for this study in 2011 and 2012, respectively. Initial sample size calculation based upon attaining a power of 80% to estimate clinically important differences in BBQ scores of 1.9 points between the two practice settings indicated a required sample size of 314, assuming a standard deviation in BBQ of 6 points, and ***α*** = 0.05. Therefore a target sample size of n = 465 was estimated, to allow for a potential 33% non-returned or incomplete questionnaires. To be included in this study, the HCPs must have had provided treatment, or cared for, patients with LBP in their clinical practice as well as having worked more than six months at their current institution. HCPs with or without LBP were included in the study. It is recognised that the sampled HCPs for this study may have varying academic and professional education backgrounds; hence, no HCPs were excluded based on their academic history, especially for older medical practitioners who may have attained a Diploma in Medicine in the earlier years of medical education in China [51].

The data were collected by local research assistants, trained for this study. The research assistants first approached the Heads of Departments of each discipline in the hospitals and community health centres to explain the purpose of the study and to seek permission to invite staff members to participate in a cross-sectional survey. Once permission was granted, the research assistants approached the HCPs at their place of work during lunchtime to canvass their interest in participation. Participant information sheets (in Simplified Chinese) were provided and the research assistants conducted a brief verbal screening to ensure that the participants met the inclusion criteria, namely their experience treating or caring for patients with LBP and length of time working in the institution, before seeking informed consent. The surveys (also in Simplified Chinese) were then distributed and the completed questionnaires were collected within a period of 24 hours.

Approval to conduct this study was granted by the Institutional Review Board of Fudan University (Shanghai, China) and Human Research Committees of Curtin University and Edith Cowan University (Perth, Western Australia). All participants gave informed consent prior to participation in this study.

### Procedure and outcome measures

Participants completed a number of questionnaires that had all been translated into Simplified Chinese using the double-back translation method previously utilised by this research team [52]. First, all participants completed a self-report questionnaire that sought personal information such as: gender, age, height, weight, occupation, number of years in their current profession, highest formal education, and history of LBP (Yes/No answer). All participants completed the Back Beliefs Questionnaire (BBQ), while only participants with a history of LBP completed the Fear Avoidance Beliefs Questionnaire (FABQ). Participants who indicated that they had previously experienced LBP were also requested to complete instruments which assessed their LBP status, severity and impact using the Nordic Musculoskeletal Questionnaire (NMQ), a Visual Analogue Scale (VAS), and the Roland Morris Disability Questionnaire (RMDQ), respectively.

### Instruments assessing back pain beliefs Back Beliefs Questionnaire (BBQ)

All participants completed the BBQ, which examines the general beliefs about the inevitable consequences of future life with LBP. This questionnaire consists of 14 statements scored on a five-point Likert scale ranging from 1 (completely disagree) to 5 (completely agree). Scores range between 9 to 45, with five statements acting as distractors. Lower scores represent more negative beliefs of an individual towards low back trouble. The BBQ has been previously shown to possess adequate internal consistency (Cronbach’s ***α*** = 0.70) and test-retest reliability (ICC = 0.87) [53]. A translation of the BBQ into Simplified Chinese has been undertaken by our research group and has been found to have good test-retest reliability (ICC = 0.85) and internal consistency (Cronbach’s ***α*** = 0.70) [52].

### Fear-Avoidance Beliefs Questionnaire (FABQ)

Participants with a history of LBP completed the FABQ, which contains two sub-scales; fear avoidance beliefs in relation to work (FABQ-work) and physical activity (FABQ-physical) [23]. The FABQ-work sub-scale has 11 questions and the FABQ-physical has five questions rated on a Likert scale from 0 (completely disagree) to 6 (completely agree). The work sub-scale scores range between 0 to 42, derived by adding 7 items (4 distractor items) and the physical sub-scale range between 0 to 24 by adding 4 items (1 distractor) [23]. A higher score indicates more strongly held fear avoidance beliefs. The internal consistency (Cronbach’s ***α***) of the FABQ-work and FABQ-physical have been reported to be 0.88 and 0.77, respectively [23]. The Simplified Chinese versions of the FABQ-work and FABQ-physical have shown a Cronbach’s ***α*** of 0.97 and 0.78, respectively and ICCs of 0.90 to 0.93 [52].

### Instruments assessing LBP status, severity and impact Modified Nordic Musculoskeletal Questionnaire about Low Back Trouble (NMQ)

In this study, the NMQ pertaining to low back trouble was used to identify the respondents who had experienced LBP in their lifetime and in the last 12 month. The test-retest reliability of the questionnaire as it relates to LBP was first reported by Kuorinka et al. [54] in 1987 where the percentage of disagreeing answers averaged 4.4%. Further iterations of the tool have also been reported to be reliable [55].

### Visual Analog Scale (VAS)

The VAS is an instrument that measures the intensity of pain. This instrument consists of a 100 mm horizontal line, anchored by word descriptors at each end, namely; “no pain” and “very severe pain”. The participants were asked to indicate their *usual level of (low back) pain in the last week* by placing a cross on the horizontal line. This method of measuring pain has been widely used and has previously been found to be reliable and valid in English [56]. There is an absence of previous psychometric evaluation of the Simplified Chinese version of this scale, possibly due to its simplicity and ubiquitous use.

### Roland-Morris Disability Questionnaire (RMDQ)

The RMDQ is a 24 item self-reported questionnaire measuring the physical disability level in people with LBP. Scores may range from 0 (no disability) to 24 (maximum disability). The RMDQ has been found to have a high degree of reliability (ICC = 0.88) and internal consistency (Cronbach’s ***α*** between 0.84-0.93) [57]. Psychometric analyses of Simplified Chinese versions of this questionnaire have been conducted and were shown to possess consistently good test-retest reliability (ICC = 0.95) and internal consistency (Cronbach’s ***α*** ranging from 0.83 to 0.88) [48,58].

### Statistical analysis

The participants were asked to indicate their *highest* formal education qualification, and these were subsequently classified as: secondary technical qualification, junior college/senior high school, bachelor’s degree, master’s degree or PhD (these latter two categories were subsequently classified as having a postgraduate education). The HCPs were also grouped according to the following occupational groups: i) nurses; ii) medical practitioners, which included general practitioners, rehabilitation medicine specialists and rehabilitation specialist trainee practitioners; iii) therapists, which included general rehabilitation therapists and therapists in specialised fields, for example, physiotherapists or occupational therapists; iv) traditional Chinese medicine practitioners category included acupuncturists who used needling and cupping as part of their treatment for low back pain; and iv) other: this group included trainee medical practitioners, trainee therapists or medical practitioners in other fields for example obstetricians or surgeons who indicated having had experience managing patients with LBP.

Participants were also classified according to their location of work; ‘metropolitan’ or ‘regional’ if they worked in one of the tertiary and teaching hospitals in metropolitan Shanghai place or in community healthcare centres in Jin Shan district, respectively.

To investigate the influence of LBP and LBP-related disability on back pain beliefs, the sample was re-classified into three groups according to their LBP status and LBP-related disability levels. The groups were classified into i) those who reported no history of LBP (No LBP group); ii) those who reported a history of LBP and a disability score of < 7 on the RMDQ (LBP-low disability group) and; iii) those with a history of LBP and a RMDQ score of ≥ 7 (LBP-high disability group). The cut-off point of 7 was used to classify ‘low’ and ‘high’ disability as it has been previously reported that people with LBP and higher disability (RMDQ scores 7 to 13) were 9.8 times more likely be off work six months after injury compared to those who had lower disability scores (RMDQ scores 0 to 6) [59]. Further, nurses with back pain reporting modified duties or work absence have previously reported a mean RMDQ score of 8 [16].

Univariable associations between the three dependent variables i) BBQ ii) FABQ-physical and ii) FABQ-work and independent variables were assessed using linear regression analysis. Three multivariable linear regression models were estimated to examine the independent associations between the three dependent variables: i) BBQ ii) FABQ-physical and ii) FABQ-work, and each independent variable adjusting for all other independent variables. BBQ models included all participants whereas FABQ models included only those participants reporting LBP. Standard regression diagnostics were assessed, including standardised residual plots, partial regression plots, and variance inflation factors. Absence of influential data points and collinearity, and normality and homoscedasticity of residuals were also confirmed. Statistical analysis was performed using Stata/IC 10.1 for Windows (Statacorp LP, College Station TX). Statistical significance was set at ***α*** = 0.05.

## Results

A total of 231 and 201 questionnaires from tertiary hospitals in metropolitan Shanghai and community health centres in Jin Shan district, respectively, were used for analysis. Of the 240 questionnaires distributed to HCPs working in tertiary hospitals, 234 questionnaires were returned and three were rejected due to multiple incomplete items. In Jin Shan district, 225 questionnaires were distributed and of these, 216 were returned. Fifteen questionnaires were further rejected due to several incomplete demographic items or no LBP status indicated.

The demographic characteristics of the various disciplines of HCPs working in metropolitan tertiary hospitals and regional community health centres in Shanghai are summarised in Table 1. 40% to 61% of all occupational groups reported having had experienced LBP in their lifetime with the exception of the five traditional Chinese medicine practitioners working in the metropolitan hospitals who indicated that they never experienced LBP. Similarly, 38.5% to 58.7% (Table 1) of all occupational groups reported having had experienced LBP in the last 12 months. Overall, the HCPs in this study reported fairly low pain levels on the VAS for *usual pain in the past week* with median scores ranging from 0.8 to 2.6 (Table 1).

**Table 1:** Demographic characteristic of healthcare professionals working in tertiary hospitals in metropolitan Shanghai (Xuhui, Jing’An and Yangpu districts) and community health centres in regional Shanghai (Jin Shan district)

**Demographic characteristic**	**Tertiary hospitals in metropolitan Shanghai (N = 231)**					**Community health centres in regional Shanghai (N = 201)**				
	**Nurses**	**Medical Practitioners**	**Therapists**	**TCMP**	**Other**	**Nurses**	**Medical Practitioners**	**Therapists**	**TCMP**	**Other**
N (%)^a^	111 (48.1)	46 (19.9)	44 (19.0)	5 (2.2)	25 (10.8)	102 (51.3)	71 (35.7)	0	13 (6.5)	13 (6.5)
Gender: n (%) female^b^	109 (98.2)	25 (54.3)	18 (40.9)	3 (30.0)	14 (56.0)	98 (97.0)	30 (42.3)	0	6 (46.2)	3 (23.1)
Highest academic qualification%^c^ Secondary technical	16.2	2.2	11.4	0	4.0	36.4	14.3	-	30.8	33.3
Junior college/Senior high	56.8	2.2	20.5	0	4.0	58.6	30.0	-	30.8	16.7
Bachelor degree	23.4	*43.5	56.8	60.0	60.0	5.1	*50.0	-	30.8	50.0
Postgraduate degree	3.6	52.2	11.4	40.0	32.0	0	5.7	-	7.7	0
Age: mean (SD)^a^	32.9 (10.0)	39.3 (10.0)	35.0 (9.7)	38.8 (8.6)	29.9 (10.8)	35.8 (9.9)	40.6 (10.2)	-	46.5 (10.6)	43.7 (9.9)
Years of practice: mean (SD)^a^	11.5 (10.1)	14.1 (10.2)	12.7 (10.5)	16 (8.7)	7.8 (11.9)	15.0 (10.1)	18.3 (10.9)	-	22.6 (10.5)	22.1 (10.1)
Lifetime experience of LBP n (%)	65 (58.6)	24 (52.2)	24 (54.5)	0 (0.0)	10 (40.0)	59 (57.8)	37 (52.1)	-	7 (53.8)	8 (61.5)
Experience of LBP in last 12 month n (%)	64 (57.7)	22 (47.8)	24 (54.5)	-	10 (40.0)	55 (53.9)	35 (49.3)	-	5 (38.5)	7 (53.8)
Usual pain last week (/10) Median (range)	1.7 (0–8)	1.2 (0–7)	2.0 (0–8)	-	1.6 (0–4)	2.1 (0–10)	1.9 (0–9)	-	2.6 (1–8)	0.8 (0–5)

TCMP = Traditional Chinese Medicine Practitioners;

*A proportion of medical practitioners do not have a bachelor’s degree. They may have undertaken a certificate-oriented three-year medical training course at a medical college before the reform in medical education in the late 1990s[51]*.*

a: n = 2 data points missing for rural practitioners, N = 199.

b: n = 3 data point missing for rural practitioners, N = 198.

c: n = 7 data point missing for rural practitioners, N = 194.

For BBQ scores, there were significant group differences in scores in univariable analysis for location of work, occupation, gender, and age (Table 2) where more positive beliefs were identified among metropolitan-based practitioners, males, and in older practitioners. Differences in beliefs between occupational groups were also identified in univariable analysis. The estimates from the multivariable model are presented in Table 3, and indicate significant independent associations between BBQ scores and location of work and age. It was estimated that HCPs working in community health centres in regional Shanghai had lower BBQ scores than HCPs working in tertiary hospitals in metropolitan Shanghai (mean difference [95% CI]: −2.4 [3.8 - −1.0], p = 0.001), and that older HCPs (>40) had higher BBQ scores than 20–29 year olds (mean difference [95% CI]: 2.4 [0.9 - 3.9], p = 0.001) and 30 to 39yr olds (mean difference [95% CI]: 1.6 [0.1- 3.1], p = 0.036). The estimated difference of lower BBQ scores in females compared to males was attenuated and not statistically significant after adjusting for other independent variables (mean difference [95% CI]: −1.4 [−3.0 - 0.2], p = 0.082). However, there were no significant differences in BBQ scores according to occupation, education, or LBP disability after adjusting for all other variables. The adjusted R^2^ for the multivariable model was 0.054.

**Table 2:** Group frequencies and group means for beliefs about low back pain for independent variable categories (N = 432)

	**N (%)**	**BBQ Mean (SD)**	**p-value**^**1**^
Location of work			
Metropolitan hospitals	231 (53.5)	29.2 (5.8)	0.003
Regional community health centres	201 (46.5)	27.4 (6.5)	
Occupation			
Nurses	213 (49.5)	27.3 (6.6)	0.014
Medical practitioners	117 (27.2)	29.4 (5.9)	
Therapists	44 (10.2)	29.7 (5.2)	
TCMP	18 (4.2)	30.1 (4.9)	
Other	38 (8.8)	28.3 (5.8)	
LBP Disability			
No LBP	196 (45.4)	28.3 (6.2)	0.492
LBP low disability	151 (34.9)	28.7 (6.1)	
LBP high disability	85 (19.7)	27.7 (6.6)	
Gender			
Male	125 (29.0)	29.9 (6.0)	0.001
Female	306 (71.0)	27.7 (6.2)	
Age			
20 to29 yrs	143 (33.1)	27.3 (5.5)	0.011
30 to 39 yrs	118 (27.3)	28.0 (6.0)	
40 yrs or greater	171 (39.6)	29.4 (6.8)	
Academic Qualification			
Secondary technical	80 (18.7)	27.9 (5.9)	0.057
Junior college/Senior high	159 (37.2)	27.6 (6.8)	
Bachelor degree	139 (32.6)	29.1 (5.6)	
Postgraduate degree	49 (11.5)	29.8 (5.9)	

^*1*^*p-value for overall test for the difference in groups means, using dummy variable coding in univariable linear regression models.*

BBQ = beliefs about low back pain derived from the Back Belief Questionnaire; scores ranged between 9–45 with lower scores representing more negative beliefs.

TCMP = Traditional Chinese Medicine Practitioners.

Other: included trainee medical practitioners, trainee therapists or medical practitioners in other fields for example obstetricians or surgeons who have had experience managing patients with LBP.

**Table 3:** Summary of the estimates from the multivariable regression model for beliefs about low back pain using the Back Belief Questionnaire (BBQ) as the dependent variable

	**Marginal Means**	**Unstandardised regression coefficient**^**1**^	**95% CI**			**p-value**
Location of work						
Metropolitan hospitals	29.5	REF				
Regional community health centres	27.1	−2.4	−3.8	-	−1.0	0.001
Occupation						
Nurses	28.1	REF				0.773^2^
Medical practitioners	29.0	0.9	−0.9	-	2.7	0.341
Therapists	28.1	0.0	−2.3	-	2.3	1.000
TCMP	29.7	1.6	−1.6	-	4.8	0.321
Other	28.1	0.0	−2.4	-	2.4	0.977
LBP Disability						
No LBP	28.5	REF				0.387^2^
LBP low disability	28.8	0.3	−1.0	-	1.6	0.650
LBP high disability	27.6	−0.9	−2.5	-	0.8	0.298
Gender						
Male	29.4	REF				
Female	28.0	−1.4	−3.0	-	0.2	0.082
Age						
20 to29 yrs	27.2	REF				0.005^2^
30 to 39 yrs	28.0	0.8	−0.8	-	2.3	0.326
40 yrs or greater	29.6	2.4^3^	0.9	-	3.9	0.001
Academic Qualification						
Secondary technical school	28.5	REF				0.951^2^
Junior college/Senior High	28.2	−0.3	−2.0	-	1.3	0.683
Undergraduate degree	28.6	0.1	−1.9	-	2.0	0.956
Postgraduate degree	28.3	−0.3	−2.9	-	2.3	0.843

^*1*^*adjusted for all other independent variables.*

^*2*^*Joint Wald test for coefficients.*

^*3*^*contrast to ’30-39 yrs’ category = 1.6(95% CI: 0.1 to 3.1, p = .036).*

TCMP = Traditional Chinese Medicine Practitioners.

Other: included trainee medical practitioners, trainee therapists or medical practitioners in other fields for example obstetricians or surgeons who have had experience managing patients with LBP.

In those participants with a history of LBP, the only variable for which a significant group difference in FABQ-physical scores was estimated in univariable analyses was for LBP disability (Table 4), where practitioners with higher LBP-related disability reported higher FABQ-physical scores. This difference remained similar after adjusting for all other variables (Table 5), with HCPs reporting highly disabling LBP estimated to have higher FABQ-physical scores than those reporting low disability (mean difference [95% CI]: 2.8 [1.5 - 4.1], p < 0.001). In the adjusted model, those with postgraduate degrees had lower scores (mean difference [95% CI]: 2.9 [−5.8 - 0.0], p = 0.049) than those with secondary/technical qualifications, although the omnibus test for group differences for this variable was not statistically significant and there were no other significant group contrasts. No other variables were estimated to have associations with FABQ-physical scores in the multivariable model. The adjusted R^2^ for the multivariable model was 0.082.

**Table 4:** Group frequencies and group means for the sub-scale Fear Avoidance Beliefs (FABQ-physical and FABQ-work sub scales) for independent variable categories (LBP only, N = 236)

	**N (%)**	**FABQ-Phys Mean (SD)**	**p-value**^**1**^	**FABQ- Work Mean (SD)**	**p-value**^**1**^
Location of work					
Metropolitan hospitals	124 (52.5)	17.9 (4.4)	.216	17.9 (9.2)	0.254
Regional community health centres	112 (47.5)	17.1 (5.2)		16.6 (7.3)	
Occupation					
Nurses	125 (53.2)	17.9 (4.7)	.427	17.5 (8.7)	0.300
Medical practitioners	61 (26.0)	16.9 (4.9)		16.8 (7.5)	
Therapists	24 (10.2)	18.1 (4.9)		20.1 (8.8)	
TCMP	7 (3.0)	15.3 (5.3)		12.5 (5.6)	
Other	18 (7.7)	17.0 (4.4)		16.4 (8.3)	
LBP Disability					
LBP low disability	151 (64.0)	16.4 (5.0)	<.001	15.2 (8.5)	<0.001
LBP high disability	85 (36.0)	19.5 (3.7)		20.9 (6.8)	
Gender					
Male	67 (28.5)	17.1 (4.8)	.414	18.2 (8.3)	0.318
Female	168 (71.5)	17.6 (4.8)		16.9 (8.4)	
Age					
20 to29 years	68 (28.8)	17.5 (4.2)	.988	17.1 (7.8)	0.421
30 to 39yrs	64 (27.1)	17.5 (4.9)		18.4 (8.4)	
40 or greater	104 (44.1)	17.4 (5.1)		16.7 (8.7)	
Academic Qualification					
Secondary technical	47 (20.1)	18.2 (5.3)	.159	17.5 (8.5)	0.596
Junior college/Senior High	89 (38.0)	17.3 (4.9)		17.2 (8.1)	
Bachelor degree	75 (32.1)	17.7 (4.4)		17.9 (8.9)	
Postgraduate degree	23 (9.8)	15.6 (4.2)		15.2 (7.2)	

^*1*^*p-value for overall test for the difference in groups means, using dummy variable coding in univariable linear regression models.*

TCMP = Traditional Chinese Medicine Practitioners.

Other: included trainee medical practitioners, trainee therapists or medical practitioners in other fields for example obstetricians or surgeons who have had experience managing patients with LBP.

**Table 5:** Summary of the estimates from the multivariable regression model for FABQ-Physical (N = 231) and FABQ-Work (N = 230)

	**FABQ-Physical**						**FABQ-Work**					
	**Marginal means**	**Unstandardised regression coefficient**^**1**^	**95% CI**			**p-value**	**Marginal means**	**Unstandardised regression coefficient**^**1**^	**95% CI**			**p-value**
Location of work												
Metropolitan hospitals	17.9	REF					17.6	REF				
Regional community health centres	16.8	−1.1	−2.6	-	0.4	0.148	17.0	−0.6	−3.2	-	2.0	0.639
Occupation												
Nurses	17.7	REF				0.781^2^	17.5	REF				0.483^2^
Medical practitioners	17.4	−0.3	−2.2	-	1.5	0.728	17.5	0.0	−3.2	-	3.2	0.996
Therapists	17.4	−0.3	−2.9	-	2.3	0.814	18.7	1.3	−3.2	-	5.7	0.572
TCMP	15.8	−1.9	−5.6	-	1.9	0.324	13.3	−4.2	−10.7	-	2.2	0.200
Other	16.4	−1.3	−4.0	-	1.3	0.325	15.3	−2.1	−6.7	-	2.4	0.359
LBP Disability												
LBP low disability	16.4	REF					15.1	REF				
LBP high disability	19.2	2.8	1.5	-	4.1	<0.001	21.3	6.2	4.0	-	8.4	<0.001
Gender												
Male	17.7	REF					19.5	REF				
Female	17.3	−0.4	−2.1	-	1.4	0.677	16.5	−3.0	−6.0	-	0.0	0.053
Age												
20 to29 yrs	17.4	REF				0.847^2^	17.6	REF				0.044^2^
30 to 39 yrs	17.7	0.4	−1.3	-	2.0	0.665	19.3	1.7	−1.1	-	4.4	0.239
40 yrs or greater	17.3	−0.1	−1.6	-	1.5	0.924	15.9	−1.7^3^	−4.4	-	0.9	0.205
Academic Qualification												
Secondary technical	18.5	REF				0.165^2^	18.0	REF				0.618^2^
Junior college/Senior High	17.2	−1.3	−3.0	-	0.4	0.137	17.6	−0.5	−3.4	-	2.4	0.753
Bachelor degree	17.7	−0.8	−2.7	-	1.2	0.446	17.4	−0.7	−4.0	-	2.7	0.689
Postgraduate degree	15.5	−2.9	−5.8	-	0.0	0.049	14.8	−3.2	−8.2	-	1.8	0.205

^*1*^*adjusted for all other independent variables.*

^*2*^*Joint Wald test for coefficients.*

^*3*^*contrast to ’30-39 yrs’ category = −3.4 (95% CI: −6.0 to −0.7, p = .013).*

TCMP = Traditional Chinese Medicine Practitioners; Other: included trainee medical practitioners, trainee therapists or medical practitioners in other fields for example obstetricians or surgeons who have had experience managing patients with LBP.

Similarly, in those participants with a history of LBP, the only variable for which significant group differences in FABQ-work scores was estimated in univariable analyses was for LBP disability (Table 4), where practitioners with higher LBP-related disability reported higher fear avoidance attitudes. This difference remained after adjusting for all other variables (Table 5), with HCPs reporting highly disabling LBP estimated to have higher FABQ-work scores than those reporting low disability (mean difference [95% CI]: 6.2 [4.0 - 8.4], p < 0.001). After adjustment for other variables, there was also a statistically significant difference in FABQ-work scores according to age, with older HCPs (>40 years of age) estimated to have lower FABQ-work scores than 30 to 39 years old (mean difference [95% CI]: −3.4, [−6.0 - −0.7], p = 0.013). Although the estimated difference of lower FABQ-work scores in females compared to males was increased after adjusting for other variables, it was not statistically significant in the multivariable model (mean difference [95% CI]: −3.0 [−6.0 - 0.0], p = 0.053). The adjusted R^2^ for the multivariable model was 0.123.

## Discussion

To our knowledge this is the first study that has investigated the LBP-related beliefs of Chinese HCPs working in metropolitan and regional China, specifically, their beliefs about the inevitable consequences of LBP and fear avoidance beliefs related to their own LBP experience. Our results suggest that younger HCPs and those working in regional community health centres had more negative beliefs about the inevitable consequences of future life with LBP. The estimated statistically significant difference of 2.4-point in BBQ scores between younger (20–29 age group) and older HCPs (>40 years old) and in different work settings is considered clinically meaningful [34]. Buchbinder and colleagues [34], in a two-year population-based longitudinal study, documented that a 2-point change in BBQ score was associated with a reduction in medical claims related to the management of LBP.

Fear avoidance beliefs were also associated with age, where younger HCPs with LBP were more fearful that engaging in work-related activities would exacerbate their LBP. HCPs who experienced greater LBP-related disability also reported greater fear avoidance attitudes with this study estimating a 3- to 6-point difference in the FABQ physical and work subscale, respectively. The minimal clinically significant changes for the FABQ subscales have not been well validated but it has been theorized that a 6-point change in the FABQ-physical will see a clinically meaningful reduction in self-perceived disability based on the Oswestry Disability Index [60].

This study estimates that age is an important correlate for both BBQ and FABQ-work scores suggesting that older HCPs, regardless of their disciplines or occupation, had more positive beliefs (better outlook) about inevitable consequence of LBP and less fear avoidance beliefs about work. Possible reasons for this may be because they have more experience in treating people with pain; they may be indifferent to their LBP and continue with their normal level of work and socialization [61]; and because of their positive beliefs, they play a more active role in their own management [62]. Further, we have captured the older HCPs who continued to stay in the workforce, due to their more positive beliefs, as those who were more fearful that work may make their LBP condition worse may have left the profession. Whilst other studies did not find age to be an important correlate of BBQ [61,63] and FABQ [30,64], direct comparisons cannot be made due to the difference in age-group classification used [30,64] and difference in the characteristics of the samples [61,63].

Healthcare professionals working in regional community health centres were estimated to have poorer beliefs about the consequences of LBP compared to those working in tertiary teaching hospitals in metropolitan Shanghai. As beliefs can be shaped by education [34,35,39], it is reasonable to suggest that the following reasons may account for this; first, there may be lack of resources and opportunities to access contemporary evidence-based clinical guidelines in the more geographically-remote regions. It has been documented that accessibility to LBP information, services and training was more limited in rural country towns compared to metropolitan areas in Western nations, and it is likely this situation is mirrored in China [65]. Second, those HCPs working in teaching hospitals may have more opportunity for continuing professional education programs, attendance at talks/seminars by international speakers and international exchanges; hence greater exposure to current evidence-based practices. Third, by virtue of co-location of clinical teams in tertiary centres, HCPs may more readily engage in interdisciplinary practice for management of LBP and therefore be exposed to more positive patient outcomes associated with this model of care compared to single-discipline practice. Fourth, the level of English amongst HCPs in Shanghai city is generally better than in the outskirts of Shanghai, which may be a barrier to accessing research literature published in English. However, the location of work was not associated with fear avoidance beliefs in HCPs with LBP. This may highlight the general work ethic of the Chinese, where they are generally not afraid of hard work nor pain and just “get on with it” [66].

In this study, back pain beliefs did not differ amongst different HCP groups unlike previous studies which used the same scales among students of different healthcare disciplines [29,35,67]. While there is evidence that back pain beliefs are different in different professional groups who treat LBP patients [31], direct comparisons cannot be made due to the use of different scales to assess these beliefs and the cross cultural differences between HCPs working in different countries.

Our study suggested that the level of academic qualification is also associated with fear avoidance beliefs. In this study, those with postgraduate degrees (master or doctoral degrees) had significantly lower FABQ-physical scores compared to those with secondary technical academic qualifications. No differences were observed in FABQ-physical scores among other educational categories, suggesting that a substantial variation in academic training is needed (secondary technical vs. postgraduate) before beliefs are mediated by education. The more positive FABQ-physical beliefs observed in postgraduate qualified HCPs may be attributed to their exposure to more contemporary evidence-based literature and/or resources as a result of their formal engagement in postgraduate studies/research which may have been attained locally or overseas.

In this study, Chinese HCPs with a history of LBP who reported higher LBP-related disability levels were estimated to be significantly more fear avoidant, a finding consistent with previous cross sectional and prospective studies which showed that fear avoidance beliefs are predictive of disability [68,69]. A prospective study involving a large cohort of female healthcare workers showed that high fear avoidance beliefs were predictive of work absenteeism [70]. The fear avoidance model proposes that when the experience of LBP is associated with negative LBP and/or fear avoidance beliefs, this can lead to avoidance of work, social or physical activities, setting up a vicious cycle of chronicity and disability [61,71,72]. Evidence points toward a biopsychosocial understanding of LBP, with a focus on fear reduction, self-management and adopting healthy lifestyle behaviours as being a more efficacious model of care of LBP [62]. An important component of this model is the beliefs system [73].

There are several studies investigating back pain beliefs and fear avoidance beliefs in general populations and in patients with chronic pain [21,24,68,69,74]. Information regarding these back pain beliefs in practicing HCPs is presently limited. Direct comparisons with previous studies are also difficult due to the lack of consistency in outcome measures [26,32,70,75].

The medical practitioners in this study had similar mean FABQ-work scores (16.8; SD 7.5) compared to French general practitioners (17.5; SD 6.7) [30] and rheumatologists (16.7; SD 6.9) [76], but Chinese medical practitioners had higher levels of fear avoidance beliefs related to physical activities than French GPs [30] (16.9; SD 4.9 vs 9.6; SD 4.8) and rheumatologists (16.9; SD 4.9 vs 9.2; SD 4.4) [76]. In contrast, a large cohort of Australian GPs (n = 2556) from various states across Australia had mean FABQ-physical scores ranging from 13.3 - 14.0, lower than the medical practitioners in this study [34]. That Australian-based study used a modified FABQ-work (summation of 6 items instead of 7 items) thus cannot be directly compared with this study [34].

Overall, the results of this study revealed that only a small proportion of variance in the dependent variables were explained in the multivariate models. This suggests that potentially more important correlates of the beliefs were not measured in this study. These factors may include the chronicity of pain [36], health literacy [24], experience in clinical practice specifically related to their frequency and exposure to the management of patients with LBP [29,40], their practice behavior (advice about work, activity and bedrest) [75], management approach to LBP and the aetiologic framework adopted to explain LBP [77], time off work due to their LBP [78] and the nature and types of formal and informal postgraduate education or professional training [34,40,73,79,80]. Future studies will need to consider these factors to gain a more comprehensive understanding of factors influencing Chinese HCPs back pain beliefs, especially those factors that are modifiable.

Additionally, due to the non-probability sampling used in this study, the results of this study may not be generalizable to all HCPs working in China. China is an expansive country with many provinces that have differing cultures, ethnic groups, socioeconomic status and health outcomes [3]. This study attempted to assess if different professional settings influenced back pain beliefs within two arbitrarily-defined geographic locations *– urban and urban–rural mix*. These hospitals and district community health centers were conveniently sampled due to an existing working relationship with the researchers involved with these sites. The convenience sampling used in this study may have also resulted in recruiting those who were more research aware or have inherent interests in LBP (being sufferers themselves). This may have accounted for the high percentage of questionnaires returned and also to potential responder bias. The total numbers of possible practicing HCPs for the various disciplines in each of the healthcare settings were not determined thus limiting the interpretation of the representativeness of this sample and any potential threats for responder bias. While the authors acknowledge that a convenience sample represents a non-random sample, and thus threatens the generalizability of the findings and increases the likelihood of responder biases, this pragmatic sampling approach was used for two reasons. First, undertaking epidemiologic research in China in a relatively unexplored area presented challenges with a multinational research team and without pre-established relationships between the researchers and the health facilities. Second, we believe this is the first study undertaken to understand the attitudes and beliefs in Chinese HCPs working in China, and thus we sought to provide pragmatically-collected pilot data in this area to develop a framework for future epidemiologic investigations.

Evidence suggests that beliefs and attitude of HCPs influence their clinical management, the patients’ beliefs and consequently treatment outcomes [27,40]. The present study was aimed at understanding the beliefs of HCPs related to their own LBP experience and their drivers, which is important for the development of professional education and supporting consumers in effective co-management. Future studies should include HCPs groups from different provinces in China and the practice behaviors of these HCPs for the management of LBP. The back beliefs and treatment outcomes of LBP patient groups in China will also need to be investigated.

## Conclusions

This study found that beliefs and attitudes among Chinese HCPs related to their own LBP experience were independently associated with several factors. Younger Chinese HCPs were estimated to hold more negative beliefs and attitudes related to LBP, while HCPs working outside tertiary hospitals were estimated to have poorer beliefs concerning the inevitable consequences of LBP. Those HCPs who experienced LBP were estimated to have higher levels of fear avoidance beliefs when experiencing high LBP-related disability and lower levels of fear avoidance beliefs if they had completed postgraduate study.

## Abbreviations

BBQ: Back Beliefs questionnaire; CI: Confidence Interval; FABQ: Fear avoidance beliefs questionnaire; HCPs: Healthcare professionals; LBP: Low back pain; NMQ: Nordic musculoskeletal questionnaire; RMDQ: Roland Morris disability questionnaire; VAS: Visual analogue scale.

## Competing interests

The authors declare no competing interests, financial or non-financial.

## Authors’ contribution

BKT was responsible for the conception and design of the study, the data collection and analysis and the preparation of the manuscript. AS was responsible for the conception and design of the study, data analysis and the preparation of the manuscript. PO and AFB were responsible for the conception and design of the study and the preparation of the manuscript. CG was responsible for the design of the study, and data collection. AMB was responsible for the data analysis and the preparation of the manuscript. All authors read and approved the final manuscript.

## Pre-publication history

The pre-publication history for this paper can be accessed here:

http://www.biomedcentral.com/1471-2474/15/255/prepub

## References

[B1] Deyo RA Mirza SK Turner JA Martin BI Overtreating chronic back pain: time to back off? J Am Board Fam Med 2009 22 1 62 68 19124635 10.3122/jabfm.2009.01.080102 PMC2729142

[B2] Martin BI Deyo RA Mirza SK Turner JA Comstock BA Hollingworth W Sullivan SD Expenditures and health status among adults with back and neck problems JAMA 2008 299 6 656 664 18270354 10.1001/jama.299.6.656

[B3] Yang G Wang Y Zeng Y Gao GF Liang X Zhou M Wan X Yu S Jiang Y Naghavi M Vos T Wang H Lopez AD Murray CJ Rapid health transition in China, 1990–2010: findings from the Global Burden of Disease Study 2010 Lancet 2013 381 9882 1987 2015 23746901 10.1016/S0140-6736(13)61097-1 PMC7159289

[B4] Vos T Flaxman AD Naghavi M Lozano R Michaud C Ezzati M Shibuya K Salomon JA Abdalla S Aboyans V Abraham J Ackerman I Aggarwal R Ahn SY Ali MK Alvarado M Anderson HR Anderson LM Andrews KG Atkinson C Baddour LM Bahalim AN Barker-Collo S Barrero LH Bartels DH Basanez MG Baxter A Bell ML Benjamin EJ Bennett D Years lived with disability (YLDs) for 1160 sequelae of 289 diseases and injuries 1990–2010: a systematic analysis for the Global Burden of Disease Study 2010 Lancet 2012 380 9859 2163 2196 23245607 10.1016/S0140-6736(12)61729-2 PMC6350784

[B5] Murray CJ Vos T Lozano R Naghavi M Flaxman AD Michaud C Ezzati M Shibuya K Salomon JA Abdalla S Aboyans V Abraham J Ackerman I Aggarwal R Ahn SY Ali MK Alvarado M Anderson HR Anderson LM Andrews KG Atkinson C Baddour LM Bahalim AN Barker-Collo S Barrero LH Bartels DH Basanez MG Baxter A Bell ML Benjamin EJ Disability-adjusted life years (DALYs) for 291 diseases and injuries in 21 regions, 1990–2010: a systematic analysis for the Global Burden of Disease Study 2010 Lancet 2012 380 9859 2197 2223 23245608 10.1016/S0140-6736(12)61689-4

[B6] Poiraudeau S Rannou F Le Henanff A Coudeyre E Rozenberg S Huas D Martineau C Jolivet-Landreau I Revel M Ravaud P Outcome of subacute low back pain: influence of patients’ and rheumatologists’ characteristics Rheumatology (Oxford) 2006 45 6 718 723 16377729 10.1093/rheumatology/kei231

[B7] Rugelj D Low back pain and other work-related musculoskeletal problems among physiotherapists Appl Ergon 2003 34 635 639 14559425 10.1016/S0003-6870(03)00059-0

[B8] Smith D Mihashi M Adachi Y Koga H Ishitake T A detailed analysis of musculoskeletal disorder risk factors among Japanese nurses J Safety Res 2006 37 2 195 200 16678854 10.1016/j.jsr.2006.01.004

[B9] Lin PH Tsai YA Chen WC Huang SF Prevalence, characteristics, and work-related risk factors of low back pain among hospital nurses in Taiwan: a cross-sectional survey Int J Occup Med Environ Health 2012 25 1 41 50 22219056 10.2478/s13382-012-0008-8

[B10] Mitchell T O'Sullivan PB Burnett AF Straker L Rudd C Low back pain characteristics from undergraduate student to working nurse in Australia: a cross-sectional survey Int J Nurs Stud 2008 45 11 1636 1644 18420208 10.1016/j.ijnurstu.2008.03.001

[B11] Sikiru L Hanifa S Prevalence and risk factors of low back pain among nurses in a typical Nigerian hospital Afr Health Sci 2010 10 1 26 30 20811521 PMC2895788

[B12] Chiou WK Wong MK Lee YH Epidemiology of low back pain in Chinese nurses Int J Nurs Stud 1994 31 4 361 368 7928124 10.1016/0020-7489(94)90076-0

[B13] June KJ Cho S-H Low back pain and work-related factors among nurses in intensive care units J Clin Nurs 2011 20 3–4 479 487 20673308 10.1111/j.1365-2702.2010.03210.x

[B14] Briggs AM Bragge P Smith AJ Govil D Straker LM Prevalence and associated factors for thoracic spine pain in the adult working population: a literature review J Occup Health 2009 51 3 177 192 19336970 10.1539/joh.k8007

[B15] Schofield D Kelly S Shrestha R Callander E Passey M Percival R The impact of back problems on retirement wealth Pain 2012 153 1 203 210 22192565 10.1016/j.pain.2011.10.018

[B16] Denis S Shannon HS Wessel J Stratford P Weller I Association of low back pain, impairment, disability & work limitations in nurses J Occup Rehabil 2007 17 2 213 226 17252204 10.1007/s10926-007-9065-4

[B17] Stewart WF Ricci JA Chee E Morganstein D Lipton R Lost productive time and cost due to common pain conditions in the US workforce JAMA 2003 290 2443 2454 14612481 10.1001/jama.290.18.2443

[B18] Arthritis and Osteoporosis Victoria A problem worth solving 2013 Elsternwick: Arthritis and Osteoporosis Victoria

[B19] Koes BW van Tulder MW Ostelo R Kim Burton A Waddell G Clinical guidelines for the management of low back pain in primary care: an international comparison Spine 2001 26 22 2504 2513 discussion 2513–2504 11707719 10.1097/00007632-200111150-00022

[B20] Walsh NE Brooks P Hazes JM Walsh RM Dreinhofer K Woolf AD Akesson K Lidgren L Standards of care for acute and chronic musculoskeletal pain: the Bone and Joint Decade (2000–2010) Arch Phys Med Rehabil 2008 89 9 1830 1845 18760171 10.1016/j.apmr.2008.04.009

[B21] Urquhart DM Bell RJ Cicuttini FM Cui J Forbes A Davis SR Negative beliefs about low back pain are associated with high pain intensity and high level disability in community-based women BMC Musculoskelet Disord 2008 9 148 18983681 10.1186/1471-2474-9-148 PMC2587466

[B22] Fritz JM George SZ Delitto A The role of fear-avoidance beliefs in acute low back pain: Relationships with current and future disability and work status Pain 2001 94 7 15 11576740 10.1016/S0304-3959(01)00333-5

[B23] Waddell G Newton M Henderson I Somerville D Main CJ A Fear-Avoidance Beliefs Questionnaire (FABQ) and the role of fear-avoidance beliefs in chronic low back pain and disability Pain 1993 52 157 168 8455963 10.1016/0304-3959(93)90127-B

[B24] Briggs AM Jordan JE Buchbinder R Burnett AF O'Sullivan PB Chua JY Osborne RH Straker LM Health literacy and beliefs among a community cohort with and without chronic low back pain Pain 2010 150 2 275 283 20603025 10.1016/j.pain.2010.04.031

[B25] Daykin AR Richardson B Physiotherapists’ pain beliefs and their influence on the management of patients with chronic low back pain Spine 2004 29 783 795 15087802 10.1097/01.brs.0000115135.19082.97

[B26] Linton SJ Vlaeyen J Ostelo R The back pain beliefs of health care providers: are we fear-avoidant? J Occup Rehabil 2002 12 223 232 12389475 10.1023/a:1020218422974

[B27] Darlow B Fullen BM Dean S Hurley DA Baxter GD Dowell A The association between health care professional attitudes and beliefs and the attitudes and beliefs, clinical management, and outcomes of patients with low back pain: a systematic review Eur J Pain 2012 16 1 3 17 21719329 10.1016/j.ejpain.2011.06.006

[B28] Buchbinder R Staples M Jolley D Doctors with a special interest in back pain have poorer knowledge about how to treat back pain Spine 2009 34 11 1218 1226 discussion 1227 19407674 10.1097/BRS.0b013e318195d688

[B29] Briggs AM Slater H Smith AJ Parkin-Smith GF Watkins K Chua J Low back pain-related beliefs and likely practice behaviours among final-year cross-discipline health students Eur J Pain 2013 17 5 766 775 23139051 10.1002/j.1532-2149.2012.00246.x

[B30] Coudeyre E Rannou F Tubach F Baron G Coriat F Brin S Revel M Poiraudeau S General practitioners’ fear-avoidance beliefs influence their management of patients with low back pain Pain 2006 124 330 337 16750297 10.1016/j.pain.2006.05.003

[B31] Pincus T Foster NE Vogel S Santos R Breen A Underwood M Attitudes to back pain amongst musculoskeletal practitioners: a comparison of professional groups and practice settings using the ABS-mp Man Ther 2007 12 2 167 175 16914363 10.1016/j.math.2006.06.005

[B32] Houben RM Vlaeyen JW Peters M Ostelo RW Wolters PM Stomp-van den Berg SG Health care providers’ attitudes and beliefs towards common low back pain: factor structure and psychometric properties of the HC-PAIRS Clin J Pain 2004 20 1 37 44 14668655 10.1097/00002508-200401000-00008

[B33] Vlaeyen JW Linton SJ Are we “fear-avoidant"? Pain 2006 124 240 241 16901646 10.1016/j.pain.2006.06.031

[B34] Buchbinder R Jolley D Wyatt M Effects of a media campaign on back pain beliefs and its potential influence on management of low back pain in general practice Spine 2001 26 2535 2542 11725233 10.1097/00007632-200112010-00005

[B35] Burnett A Sze CC Tam SM Yeung KM Leong M Wang W Tan BK O'Sullivan P A cross-cultural study of the back pain beliefs of female undergraduate healthcare students Clin J Pain 2009 25 1 20 28 19158542 10.1097/AJP.0b013e3181805a1e

[B36] Briggs AM Jordan JE O'Sullivan PB Buchbinder R Burnett AF Osborne RH Straker LM Individuals with chronic low back pain have greater difficulty in engaging in positive lifestyle behaviours than those without back pain: an assessment of health literacy BMC Musculokelet Disord 2011 12 161 10.1186/1471-2474-12-161 PMC3155909 21756363

[B37] Briggs AM Slater H Bunzli S Jordan JE Davies SJ Smith AJ Quintner JL Consumers’ experiences of back pain in rural Western Australia: access to information and services, and self-management behaviours BMC Health Serv Res 2012 12 357 23057669 10.1186/1472-6963-12-357 PMC3494578

[B38] Slater H Briggs AM Bunzli S Davies SJ Smith AJ Quintner JL Engaging consumers living in remote areas of Western Australia in the self-management of back pain: a prospective cohort study BMC Musculokelet Disord 2012 13 69 10.1186/1471-2474-13-69 PMC3439262 22578207

[B39] Main CJ Waddell G Waddell G Beliefs about back pain The back pain revolution 2004 London: Churchill Livingstone 221 240

[B40] Main CJ Foster N Buchbinder R How important are back pain beliefs and expectations for satisfactory recovery from back pain? Best Pract Res Clin Rheumatol 2010 24 2 205 217 20227642 10.1016/j.berh.2009.12.012

[B41] Chan B Parker G Some recommendations to assess depression in Chinese people in Australasia Aust N Z J Psychiatry 2004 38 141 147 14961932 10.1080/j.1440-1614.2004.01321.x

[B42] Chun KM Chesla CA Cultural issues in disease management for Chinese Americans with type 2 diabetes Psych Health 2004 19 767 785

[B43] Jones RS Chow TW Gatz M Asian Americans and Alzheimer's disease: assimilation, culture, and beliefs J Aging Stud 2006 20 11 25

[B44] Fang ZZ Potential of China in global nurse migration Health Serv Res 2007 42 3 Pt 2 1419 1428 17489923 10.1111/j.1475-6773.2007.00717.x PMC1955377

[B45] Jin K Sorock GS Courtney T Liang Y Yao Z Matz S Ge L Risk factors for work-related low back pain in the People's Republic of China Int J Occup Environ Health 2000 6 1 26 33 10637534 10.1179/oeh.2000.6.1.26

[B46] Jin K Sorock GS Courtney TK Prevalence of low back pain in three occupational groups in Shanghai, People's Republic of China J Safety Res 2004 35 1 23 28 14992843 10.1016/j.jsr.2003.11.002

[B47] Barrero LH Hsu YH Terwedow H Perry MJ Dennerlein JT Brain JD Xu X Prevalence and physical determinants of low back pain in a rural Chinese population Spine 2006 31 23 2728 2734 17077743 10.1097/01.brs.0000244583.35982.ea

[B48] Yi H Ji X Wei X Chen Z Wang X Zhu X Zhang W Chen J Zhang D Li M Reliability and validity of Simplified Chinese version of Roland-Morris questionnaire in evaluating rural and urban patients with low back pain PLoS One 2012 7 1 e30807 22303457 10.1371/journal.pone.0030807 PMC3267758

[B49] Wang J, Ma J Shanghai Statistical Yearbook 2013 1994–2013 China Academic Journal Electronic Publishing House http://www.cnki.net

[B50] Wang Y 2012 Jin Shan Yearbook: Jin Shan Overview 2012 1994–2013 China Academic Journal Electronic Publishing House http://www.cnki.net

[B51] Schwarz MR Wojtczak A Zhou T Medical education in China's leading medical schools Med Teach 2004 26 3 215 222 15203497 10.1080/01421590310001642939

[B52] Chen G Tan BK Jia HL O'Sullivan P Burnett A Questionnaires to examine Back Pain Beliefs held by health care professionals: a psychometric evaluation of Simplified Chinese versions Spine 2011 36 18 1505 1511 21240047 10.1097/BRS.0b013e3181f49eec

[B53] Symonds TL Burton AK Tillotson KM Main CJ Do attitudes and beliefs influence work loss due to low back trouble? Occup Med (Lond) 1996 46 25 32 8672790 10.1093/occmed/46.1.25

[B54] Kuorinka I Jonsson B Kilbom A Vinterberg H Biering-Sorensen F Andersson G Jorgensen K Standardised Nordic questionnaires for the analysis of musculoskeletal symptoms Appl Ergon 1987 18 3 233 237 15676628 10.1016/0003-6870(87)90010-x

[B55] Dawson AP Steele EJ Hodges PW Stewart S Development and test-retest reliability of an extended version of the Nordic Musculoskeletal Questionnaire (NMQ-E): a screening instrument for musculoskeletal pain J Pain 2009 10 5 517 526 19345154 10.1016/j.jpain.2008.11.008

[B56] Ogon M Krismer M Sollner W Kantner-Rumplmair W Lampe A Chronic low back pain measurement with visual analogue scales in different settings Pain 1996 64 3 425 428 8783305 10.1016/0304-3959(95)00208-1

[B57] Roland M Fairbank J The Roland-Morris disability questionnaire and the Oswestry disability questionnaire Spine 2000 25 3115 3124 11124727 10.1097/00007632-200012150-00006

[B58] Fan SW Hu ZA Hong H Zhao FD Cross-cultural adaptation and validation of Simplified Chinese version of the Roland-Morris Disability Questionnaire Spine (Phila Pa 1976) 2012 37 10 875 22020608 10.1097/BRS.0b013e31823b0460

[B59] Turner JA Franklin G Fulton-Kehoe D Sheppard L Wickizer TM Wu R Gluck JV Egan K Worker recovery expectations and fear-avoidance predict work disability in a population-based workers’ compensation back pain sample Spine 2006 31 6 682 689 16540874 10.1097/01.brs.0000202762.88787.af

[B60] George SZ Fritz JM McNeil DW Fear-avoidance beliefs as measured by the fear-avoidance beliefs questionnaire: change in fear-avoidance beliefs questionnaire is predictive of change in self-report of disability and pain intensity for patients with acute low back pain Clin J Pain 2006 22 197 203 16428956 10.1097/01.ajp.0000148627.92498.54

[B61] Smith AJ O'Sullivan PB Beales D Straker L Back pain beliefs are related to the impact of low back pain in 17-year-olds Phys Ther 2012 92 10 1258 1267 22745197 10.2522/ptj.20110396

[B62] Chou R Huffman LH American Pain S, American College of P Nonpharmacologic therapies for acute and chronic low back pain: a review of the evidence for an American Pain Society/American College of Physicians clinical practice guideline Ann Intern Med 2007 147 7 492 504 17909210 10.7326/0003-4819-147-7-200710020-00007

[B63] Bowey-Morris J Davis S Purcell-Jones G Watson PJ Beliefs about back pain: results of a population survey of working age adults Clin J Pain 2011 27 3 214 224 21178597 10.1097/AJP.0b013e3181ffc00b

[B64] Coudeyre E Tubach F Rannou F Baron G Coriat F Brin S Revel M Poiraudeau S Fear-avoidance beliefs about back pain in patients with acute LBP Clin J Pain 2007 23 720 725 17885352 10.1097/AJP.0b013e31814da407

[B65] Slater H Briggs AM Smith AJ Bunzli S Davies SJ Quintner JL Implementing Evidence-Informed Policy into Practice for Health Care Professionals Managing People with Low Back Pain in Australian Rural Settings: A Preliminary Prospective Single-Cohort Study Pain Med 2014 doi:10.1111/pme.12351. [Epub ahead of print] 10.1111/pme.12351 24433536

[B66] Rarick CA Confucius on Management: Understanding Chinese Cultural Values and Managerial Practices J Int Manag Stud 2007 August 22 28

[B67] Ferreira PH Ferreira ML Latimer J Maher CG Refshauge K Sakamoto A Garofalo R Attitudes and beliefs of Brazilian and Australian physiotherapy students towards chronic back pain: A cross-cultural comparison Physiother Res Int 2004 9 13 23 15132024 10.1002/pri.296

[B68] Sions JM Hicks GE Fear-avoidance beliefs are associated with disability in older American adults with low back pain Phys Ther 2011 91 4 525 534 21350033 10.2522/ptj.20100131 PMC3070921

[B69] Camacho-Soto A Sowa GA Perera S Weiner DK Fear avoidance beliefs predict disability in older adults with chronic low back pain PM R 2012 4 7 493 497 22516436 10.1016/j.pmrj.2012.01.017 PMC3917604

[B70] Jensen JN Karpatschof B Labriola M Albertsen K Do fear-avoidance beliefs play a role on the association between low back pain and sickness absence? A prospective cohort study among female health care workers J Occup Environ Med 2010 52 1 85 90 20042878 10.1097/JOM.0b013e3181c95b9e

[B71] Rainville J Smeets RJ Bendix T Tveito TH Poiraudeau S Indahl AJ Fear-avoidance beliefs and pain avoidance in low back pain–translating research into clinical practice Spine J 2011 11 9 895 903 21907633 10.1016/j.spinee.2011.08.006

[B72] Vlaeyen JW Linton SJ Fear-avoidance model of chronic musculoskeletal pain: 12 years on Pain 2012 153 6 1144 1147 22321917 10.1016/j.pain.2011.12.009

[B73] Domenech J Sanchez-Zuriaga D Segura-Orti E Espejo-Tort B Lison JF Impact of biomedical and biopsychosocial training sessions on the attitudes, beliefs, and recommendations of health care providers about low back pain: a randomised clinical trial Pain 2011 152 11 2557 2563 21917377 10.1016/j.pain.2011.07.023

[B74] Kovacs F Abraira V Cano A Royuela A Gil del Real MT Gestoso M Mufraggi N Muriel A Zamora J Fear avoidance beliefs do not influence disability and quality of life in Spanish elderly subjects with low back pain Spine 2007 32 19 2133 2138 17762816 10.1097/BRS.0b013e318145a74b

[B75] Bishop A Foster NE Thomas E Hay EM How does the self-reported clinical management of patients with low back pain relate to the attitudes and beliefs of health care practitioners? A survey of UK general practitioners and physiotherapists Pain 2008 135 1–2 187 195 18206309 10.1016/j.pain.2007.11.010 PMC2258319

[B76] Poiraudeau S Rannou F Baron G Le Henanff A Coudeyre E Rozenberg S Huas D Martineau C Jolivet-Landreau I Garcia-Macé J Revel M Ravaud P Fear-avoidance beliefs about back pain in patients with subacute low back pain Pain 2006 124 305 311 16740362 10.1016/j.pain.2006.04.019

[B77] Kent PM Keating JL Buchbinder R Searching for a conceptual framework for nonspecific low back pain Man Ther 2009 14 4 387 396 18793868 10.1016/j.math.2008.07.003

[B78] Gross DP Ferrari R Russell AS Battié MC Schopflocher D Hu RW Waddell G Buchbinder R A population-based survey of back pain beliefs in Canada Spine 2006 31 2142 2145 16915103 10.1097/01.brs.0000231771.14965.e4

[B79] Buchbinder R Jolley D Improvements in general practitioner beliefs and stated management of back pain persist 4.5 years after the cessation of a public health media campaign Spine 2007 32 E156 E162 17334277 10.1097/01.brs.0000256885.00681.00

[B80] George SZ Teyhen DS Wu SS Wright AC Dugan JL Yang G Robinson ME Childs JD Psychosocial education improves low back pain beliefs: results from a cluster randomized clinical trial (NCT00373009) in a primary prevention setting Eur Spine J 2009 18 7 1050 1058 19418075 10.1007/s00586-009-1016-7 PMC2899593

